# Integration of Three-dimensional Visualization Reconstruction Technology with Problem-based Learning in the Clinical Training of Resident Physicians Specialized in Pheochromocytoma

**DOI:** 10.2174/0115734056327236250101052226

**Published:** 2025-03-07

**Authors:** Dong Wang

**Affiliations:** 1 Department of Urology, Peking Union Medical College Hospital, Beijing 100730, China

**Keywords:** Clinical teaching, PBL teaching, Pheochromocytoma, Resident physicians, Computer-assisted image processing, (3D) visualization

## Abstract

**Objective:**

We examined the effectiveness of integrating three-dimensional (3D) visualization reconstruction technology with Problem-Based Learning (PBL) in the clinical teaching of resident physicians focusing on pheochromocytoma.

**Methods:**

Fifty resident physicians specializing in urology at Peking Union Medical College Hospital were randomly divided into two groups over the period spanning January 2022 to January 2024: an experimental group and a control group. The experimental group underwent instruction utilizing a pedagogical approach that integrated 3D visualization reconstruction technology with PBL, while the control group used a traditional teaching model. A comparative analysis of examination performance and teaching satisfaction between both groups of resident physicians was conducted to assess the efficacy of the integrated 3D visualization and PBL teaching methods in clinical instruction.

**Results:**

The experimental group demonstrated superior performance in both theoretical assessment and clinical skills evaluation, along with heightened levels of teaching satisfaction compared to the control group, with statistically significant differences (*p* < 0.05). Additionally, the experimental group exhibited markedly higher scores in both theoretical examinations and practical assessments compared to their counterparts in the control group (*p* < 0.05). The results of theoretical examinations for the experimental group and the control group were 92.15±3.22 and 81.09±4.46, respectively (< 0.0001). The results of practical examinations for the experimental group and the control group were 90.17±3.48 and 70.75±5.11, respectively (< 0.0001).

**Conclusion:**

In the clinical teaching of training resident physicians specializing in urology for the management of pheochromocytoma, the integration of 3D visualization reconstruction technology with the PBL method significantly enhanced the teaching efficacy, improving both the quality of instruction and the level of satisfaction among participants.

## 1 BACKGROUND/INTRODUCTION

Pheochromocytoma (PCC) represents a rare endocrine tumor originating from the adrenal medulla. Currently, the primary treatment for PCC is surgical resection. However, the intricate positioning of the tumor and its complex interplay with adjacent organs and major vasculature render surgical intervention formidable [1].

Traditional imaging techniques, such as computed tomography (CT) and magnetic resonance imaging (MRI), provide two-dimensional anatomical images of the tumor. Estimating the three-dimensional morphology of PCC and constructing its spatial relationships from these conventional images pose significant challenges for resident physicians [2]. Furthermore, reliance on lecture-based learning (LBL), characterized predominantly by didactic instruction from educators and passive learning by students, engenders limited engagement and enthusiasm among students [3]. The rarity and complexity of PCC cases further complicate the understanding of relevant diagnostic and treatment strategies encountered by resident physicians.

The vivid demonstration of the three-dimensional images of PCC and its surrounding anatomical relationships to resident physicians, combined with scientific and effective teaching methods, could significantly enhance teaching efficacy. Three-dimensional visualization reconstruction technology simulates the three-dimensional shape of the tumor. It allows for precise analysis of the anatomical relationships of surrounding tissues through rotation, translation, and other manipulations. This capability contributes to the formulation of surgical strategies characterized by enhanced precision and safety, thereby bolstering the effectiveness and risk mitigation associated with operative interventions [1]. Furthermore, this technological modality facilitates the comprehension of the diagnostic and treatment processes of PCC among resident physicians. Problem-Based Learning (PBL) represents an interactive, student-led, teacher-supported seminar-based teaching method focused on problem-solving. It actively engages students, fosters engagement, and serves as an innovative and efficacious pedagogical approach [4, 5].

In this study, we implemented a combined approach integrating 3D visualization reconstruction technology with PBL pedagogy, utilizing it within the framework of clinical instruction for resident physicians specializing in PCC surgery at Peking Union Medical College Hospital. This combined teaching model was compared with traditional teaching methods to assess its efficacy in clinical teaching.

## 2 METHODOLOGY

### 2.1 General Information and Methods

#### 2.1.1 Research Participants

Fifty urology physicians specializing at Peking Union Medical College Hospital from January 2022 to January 2024 were selected as participants for instructional purposes. All participants in the study are required to meet the following requirements: (1) Complete standardized training for surgical residents; (2) passed the admission examination of PUMCH (Peking Union Medical College Hospital); (3) possess the title of attending physician. Employing a randomized allocation method facilitated by a random number table, they were divided into two groups, each comprising 25 individuals. The experimental group was taught using an integrated PBL and 3D visualization teaching model, while the control group used the traditional LBL teaching model. Analysis revealed no statistically significant differences in gender, age, or educational background between the two groups of resident physicians (*p* > 0.05), thereby ensuring comparability, as shown in Table **1**.

#### 2.1.2 Teaching Methods

**Fig. (1):**
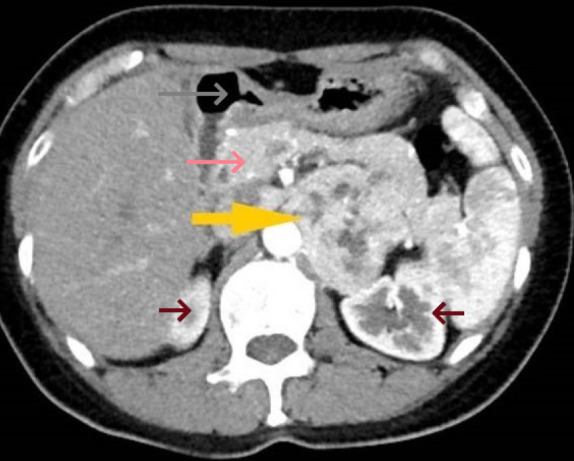
Two-dimensional CT Image of Pheochromocytoma (the tumor is indicated by a yellow arrow, the gastrointestinal tract is indicated by a brown arrow, the kidney is indicated by a purple arrow, and the pancreas is indicated by a pink arrow).

Both groups were instructed by the same senior attending physician, with each group receiving a total of 5 academic hours of instruction, each hour spanning 60 minutes. The control group adopted a traditional teaching approach. (1) Instruction was facilitated through the utilization of multimedia courseware by the teaching physician. The curriculum encompassed topics pertaining to epidemiology, etiology, clinical manifestations, diagnostic modalities, differential diagnosis, and therapeutic interventions relevant to PCC. (2) Prior to the instructional session, resident physicians were initially asked to inquire about patient histories at the bedside and review two-dimensional enhanced CT scans of the abdominal and pelvic regions (Fig. **1**) for specific case scenarios. Subsequently, the teaching physician conducted discussions on the diagnostic approach, surgical strategies, and potential postoperative complications associated with individual cases of PCC.

**Fig. (2):**
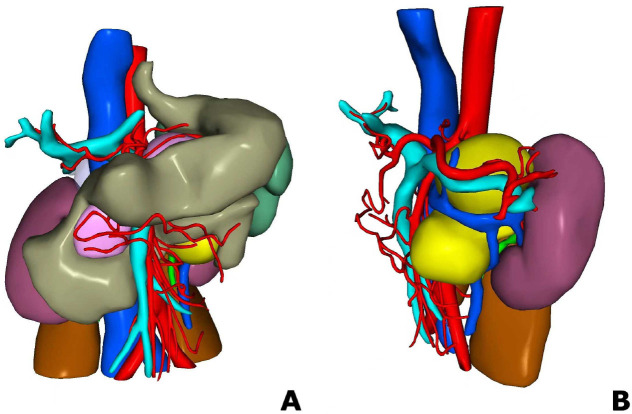
Three-dimensional reconstructed image of pheochromocytoma. (**A**): After 3D reconstruction, the approximate location of the tumor is visible (the yellow part indicates the tumor,the brown part indicates the gastrointestinal tract, and the purple part indicates the kidneys, and pink part indicates the pancreas). (**B**): Using Vitaworks software to hide and split abdominal organs, the 3D image clearly shows the tumor and its anatomical relationships with surrounding organs and blood vessels.

The experimental group used 3D visualization reconstruction technology combined with the PBL teaching method. The detailed steps are listed below:

(1) Two days before the class and the instructional session, the students received specific case materials pertinent to PCC.

(2) Resident physicians installed the “Vitaworks” 3D anatomy software on their mobile phones. Prior to the class, the teaching physician-assisted the resident physicians in learning and becoming familiar with the software's operation. Through the “Vitaworks” anatomy software, the resident physicians accessed detailed 3D reconstructed images of the specific PCC and its contiguous anatomical structures, including adjacent organs and major blood vessels (Fig. **2**). They utilized post-processing features such as measurement and segmentation to comprehensively illustrate the spatial relationships between the tumor and surrounding tissue structures, thereby enhancing their understanding of the tumor's adjacency relationships and spatial stereoscopic perception.

(3) The teaching physician posed relevant questions (*e.g*., Is the preoperative diagnosis of PCC clear? Is the preoperative fluid expansion preparation adequate? Which surgical approach—open surgery or laparoscopy—is appropriate for the case? Under what circumstances during laparoscopic surgery might a transition to open surgery be warranted? How should postoperative complications be managed?). Resident physicians discussed these questions in relation to the specific cases and reached conclusions. Subsequently, guided by the 3D reconstructed images, the teaching physician explained the diagnosis, surgical procedures, and postoperative management of the specific PCC case.

### 2.3 Observation Indicators

After the learning sessions, a unified test was conducted for all resident physicians, divided into three parts: a questionnaire-based satisfaction survey, a theoretical examination, and a practical operation evaluation.

(1) The questionnaire included 10 multiple-choice questions addressing aspects including satisfaction with the teaching method, understanding of the content, ability to stimulate initiative in learning, enhancement of clinical reasoning and problem-solving decision-making skills, proficiency in PCC diagnosis, differential diagnosis, and treatment principles, utility for future clinical practice, effective use of preoperative imaging data, determination of optimal surgical resection methods (open/laparoscopic/ conversion to open), ability to communicate surgical risks and precautions to patients, and ability to explain the specific surgical treatment plans to patients. Each question was allocated 10 points, for a total of 100 points.

(2) The theoretical examination consisted of 10 major questions based on the urology intermediate physician qualification assessment standards formulated by the instructing physician. Each question was allocated 10 points, for a total of 100 points. The examination primarily covered topics such as PCC diagnosis, differential diagnosis, auxiliary examinations, anatomy of the adrenal gland, spatial relationships between the tumor and pertinent surrounding anatomical structures and major vasculature, as well as treatment methods and management of complications.

(3) The practical operation assessment was conducted through a simulated surgical operation in urology, with the instructing physician scoring the resident physicians on criteria such as adherence to strict aseptic procedures during the surgery, precise localization of the PCC, and collaboration within the surgical team. A maximum of 100 points was attainable in this evaluation.

### 2.4 Statistical Methods

The data for this study underwent analysis utilizing SPSS statistical software version 22.0. Quantitative data are expressed as mean ± standard deviation (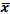 ± s) and analyzed using the t-test, while count data are presented as n (%) and analyzed using the chi-squared (χ^2^) test. A *P*-value of less than 0.05 was considered statistically significant.

## 3 RESULTS

### 3.1 Questionnaire Satisfaction Results

In the questionnaire survey assessing satisfaction among resident physicians, both groups achieved a 100% response rate. Results revealed that the experimental group scored higher in comparison to the control group, with a statistically significant difference (*p* < 0.05). Details are provided in Table **2**.

### 3.2 Theoretical Examination and Practical Operation Results

Scores obtained in both the theoretical examination and practical operation assessment were higher among resident physicians in the experimental group compared to those in the control group (Table **3**), with statistically significant differences (*p* < 0.001).

## 4 DISCUSSION

Pheochromocytoma, typically located in the retroperitoneal region, frequently demonstrates expansive growth, often exceeding diameters of 10 cm. Such large tumors frequently blur the boundaries with surrounding tissues and have a significant space-occupying effect. They may encase critical anatomical structures such as the abdominal aorta, inferior vena cava, or renal vessels, thereby substantially complicating surgical intervention [6]. The advent of 3D visualization reconstruction technology presents a promising solution to this challenge by using this innovative technology to transform two-dimensional image data into comprehensive three-dimensional models of human anatomy. This facilitates a more intuitive and thorough understanding of the patient's anatomical characteristics, thereby enabling the formulation of more precise and safer surgical plans, ultimately enhancing procedural safety and efficacy [1, 7]. Additionally, the incorporation of 3D human anatomy software has brought about significant advancements in medical education and research. It has been widely applied in clinical teaching across various surgical disciplines, including neurosurgery, general surgery, orthopedics, and obstetrics and gynecology, achieving excellent results [2, 8-11]. Endowed with the capability to generate realistic 3D images, 3D human anatomy software facilitates multifaceted manipulation, including rotation, scaling, and virtual dissection of adjacent anatomical structures, thereby fostering a comprehensive comprehension of human anatomy among students [3]. Furthermore, PBL teaching encourages learners to focus on problems, acquire knowledge related to those problems, and thereby cultivate analytical and problem-solving competencies [12].

This study represents the first implementation of 3D visualization reconstruction technology integrated with the PBL method in the clinical teaching of resident physicians specializing in PCC. Using the Vitaworks software, physicians can not only transform two-dimensional CT images into 3D reconstructions but also harness its 3D visualization technology to manipulate the resultant models through operations such as rotation, scaling, sectioning, and concealing. These capabilities allow students to make more intuitive observations of the tumor's location and its relational dynamics with adjacent anatomical structures, thereby enhancing their understanding of the surgical plan and the procedural intricacies themselves. Additionally, 3D visualization technology can be used in doctor-patient communication, aiding physicians in better explaining the condition and treatment options to patients [13]. Survey findings from this study indicate that resident physicians who received training using 3D imaging reconstruction technology combined with PBL teaching exhibited heightened levels of learning satisfaction. This teaching model promotes self-directed learning initiatives and encourages discussions among resident physicians as well as between instructors and resident physicians, making this interactive learning approach intellectually stimulating and engaging for learners. Furthermore, the vivid graphical representations provided by 3D imaging reconstruction technology enhance the teaching of PCC by helping resident physicians better understand the peripheral anatomical relationships of PCC, thereby further increasing their learning interest [3]. Resident physicians in the experimental group also widely integrated 3D imaging reconstruction technology into doctor-patient communication, facilitating a better understanding of the patient's condition and treatment options among patients and their families. Consequently, resident physicians in the experimental group exhibited a more proactive involvement in the diagnostic and treatment processes of patients with PCC.

Furthermore, the integration of 3D imaging reconstruction technology into PBL teaching facilitates the vivid depiction of rare and abstract two-dimensional PCC images. This enhances the comprehension of resident physicians regarding the distinctive characteristics of PCC cases and their diagnosis and treatment plans, thereby improving their theoretical examination scores. Additionally, 3D human anatomy software reconstructs images that closely represent authentic surgical scenarios. This software allows for the manipulation of these 3D images through rotation, scaling from diverse perspectives, and virtual dissection of adjacent organs. These features significantly enhance the resident physicians' understanding of PCC surgical procedures, thus improving their performance in practical surgical procedures.

However, the study is subject to certain limitations. Significantly, the limited sample size employed in the research and the brief duration of follow-up hinder a thorough assessment of the enduring efficacy of teaching methods over the long term. Furthermore, our integration of 3D reconstructed images with PBL teaching strategies is still at an early developmental phase, highlighting the necessity for further refinement and optimization. Therefore, it is hoped that in future clinical teaching in urology, the combination of 3D reconstructed images and the PBL method will be more widely applied to various diseases and different educational levels and that the teaching efficacy will undergo long-term evaluation.

## CONCLUSION

In conclusion, the results of this study underscore the substantive enhancement in clinical teaching efficacy observed within the domain of urology resident physician education for PCC, facilitated by the integration of 3D reconstruction technology and the PBL method.

## Figures and Tables

**Table 1:** General information about resident physicians.

**Group**	**Gender (number, Male or Female)**	**Age (years, 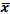 ±s)**	**Educational Background (Number, Master's or Bachelor's Degree)**
**Experimental Group**	18/7	29.1±3.5	16/9
**Control Group**	21/4	30.4±4.5	19/6
***X^2^ / t*-value**	1.048	1.140	0.857
***p*-value**	0.305	0.259	0.354

**Table 2:** Comparison of questionnaire satisfaction survey results [n (%)].

**Survey Item**	**Experimental Group (n = 25)**	**Control Group (n = 25)**	***X^2^* -value**	***p*-value**
**Satisfied with the teaching method?**	(92) 23	(64) 16	4.19	0.040
**Satisfied with the teaching content?**	(92) 23	(76) 19	1.34	0.247
**Stimulates initiative in learning?**	(96) 24	(56) 14	8.88	0.002
**Improves clinical thinking and problem-solving decision-making?**	(88) 22	(52) 13	6.09	0.013
**Mastery of diagnosis, differential diagnosis, and treatment principles of PCC?**	(96) 24	(72) 18	5.35	0.020
**Helpful for future clinical work?**	(92) 23	(60) 15	5.3	0.020
**Effective use of preoperative imaging data?**	(96) 24	(60) 15	5.37	0.020
**Determination of surgical methods?**	(84) 21	(40) 10	8.48	0.003
**Proactively inform patients about surgery risks.**	(100) 25	(72) 18	5.98	0.014
**Explains a specific surgical treatment plan to patients.**	(96) 24	(48) 12	12.00	<0.001

**Table 3:** Results of theoretical and practical examinations for both groups of resident physicians.

**Group**	**Theoretical Examination n = 25, ( 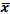 ±s)**	**Practical Operation Assessment n = 25 ( 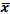 ±s)**
**Experimental Group**	92.15±3.22	90.17±3.48
**Control Group**	81.09±4.46	70.75±5.11
**t-value**	11.993	13.468
***p*-value**	< 0.0001	< 0.0001

## Data Availability

The datasets used and/or analyzed during the current study are available from the corresponding author [D.W] upon reasonable request.

## LIST OF ABBREVIATIONS

PBL: Problem-Based Learning;
PCC: Pheochromocytoma
CT: Computed Tomography
MRI: Magnetic Resonance Imaging
LBL: Lecture-based Learning

## STANDARDS OF REPORTING

STROBE guidelines were followed.
